# Synergetic effect of *Camellia sinensis* waste extract and zinc oxide nanoparticle for improving performance and appearance attributes of viscose fabrics

**DOI:** 10.1038/s41598-026-42384-4

**Published:** 2026-03-27

**Authors:** Shahd Rasmy, Salwa Mowafi, Mahmoud Suleyman, Hosam El-Sayed

**Affiliations:** 1 Faculty of Biotechnology, MSA University, 6Th of October City, Giza 12451 Egypt; 2 Proteinic and Man-Made Fibers Department, Textile Research and Technology Institute, National Research Centre, Dokki, Giza 12622 Egypt

**Keywords:** Viscose, Fabric, Black tea, Waste, Zinc oxide, Nanoparticles, Antimicrobial, Dyeing, Antioxidant, Eco-friendly, Polyphenols compounds, Biotechnology, Chemistry, Environmental sciences, Materials science, Nanoscience and technology

## Abstract

Eco-friendly textile dyeing technologies are increasingly popular because of raised environmental consciousness and the need for less polluting substitutes wefor synthetic dyes. Waste of black tea is an appropriate source of polyphenols and tannins with ecological as well as functional benefits, such as microbial resistance, and antioxidant activity. Herein, a new method for eco-friendlier dyeing and finishing of viscose fabric using black tea waste extract (BTWE) as a sustainable natural colorant for dyeing and functional finishing of viscose fabric was examined. The dyeing conditions, like pH, temperature, dye concentration, and time, were systematically regulated to assign the proper conditions for maximum color strength (K/S). The colorfastness of the dyed fabric against washing, perspiration, crocking, and light was determined. Using zinc oxide nanoparticles (ZnO-NPs) improved the performance of the dyed fabrics by making them more resistant to some pathogens. The results revealed that the optimum dyeing conditions were found to be pH 3, 45 °C, 4% dye, for 60 min. Finishing the dyed samples with ZnO-NPs enhanced the K/S and antimicrobial activity without negative impact on the mechanical strength. The dyed viscose fabric exhibited excellent resistance towards Gram-positive (*Staphylococcus aureus*) *and* Gram-negative (*Escherichia coli*) bacteria as well as the fungus *Candida albicans*. The antioxidant activity of the dyed samples was greatly improved compared to undyed viscose fabric. However, enhancement of the ultraviolet protection factor of the dyed fabric was limited, likely due to inadequate surface coverage and lack of coating operations. The discrepancy in the chemical and morphological structures between the undyed and the corresponding dyed viscose fabrics was monitored using Fourier transform infrared spectroscopy and scanning electron microscopy, respectively. The correlation between the different dyeing parameters and the color strength as well as the colorimetric data was assigned using analysis of variance (ANOVA). The findings of this investigation justify the potential application of BTWE as a green colorant for one-pot sustainable dyeing and functional finishing of viscose fabrics.

**Supplementary Information:**

The online version contains supplementary material available at 10.1038/s41598-026-42384-4.

## Introduction

Viscose is a regenerated fabric made from wood pulp that can be fabricated into fibers with a market share close to 80% of all man-made cellulose-based fibers^1^. It is a relatively inexpensive fabric, often used for its rich, vivid colors, silk-like appearance, and exceptional drape and feel. Viscose shares several characteristics with natural fibers such as cotton, such as softness, breathability, high moisture absorption, and ease of dyeing^2^. Because of their controlled physical characteristics and flexibility, viscose fibers are used in the textile and clothing sectors as well as in technical applications. However, viscose fibers have some limitations, such as low tensile strength, especially when wet; a high wrinkle-prone nature; poor heat resistance; and the lack of adequate resistance to microbial attack^3,4^.

Dyeing and finishing of textile substrates are carried out to improve the appearance and performance attributes of the final textile products^5–7^. Some synthetic and natural colorants can improve particular performance characteristics of textile fabrics, such as microbial resistance^8^, insect-proofing^9^, and ultraviolet protection^10^. Traditionally, synthetic dyes, such as anionic, cationic, reactive, direct, disperse, and vat dyes, have been extensively used in dyeing and printing textile substrates, including viscose fibers^11–13^. Sizing, desizing, scouring, and bleaching are some of the procedures used before viscose dyeing to improve dye absorption and ensure coloration^14^.

Reactive dyes furnish a wide gamut of shades of good lightfastness and excellent wash fastness on textile fibers, making them a more desired class of dye in the textile industry^15,16^. Direct dyes are easy to use for coloration of cellulosic fibers, but their wash-fastness performance is only moderate^17^.Vat dyes are water-insoluble dyes, typically based on anthraquinone or indigo structures, and are used to dye cellulosic materials through a vat dyeing process^18^. Improper disposal of synthetic dyes in the textile sector would harm the environment, including the air, water, soil, and plants. Dyes have a negative impact on aquatic environments because they reduce light penetration, which limits algae growth that is required for oxygen production and the aquatic food chain. Additionally, dyes may induce allergic reactions such as skin and eye irritation, dermatitis, and respiratory problems^19^.

As a safer and more glorious alternative, natural colorants have become widely used in the textile industry for dyeing and printing various textiles, food substrates, paper, leather, and cosmetics^20,21^. In recent years, natural dyes have received increasing attention due to their eco-friendliness and multifunctional characteristics. As a result, they now hold a considerable share in the dye market for textiles, especially natural fibers^22,23^. However, the complex structures of natural dyes, diversity in sourcing materials, and difficulties in controlling dyeing conditions make it difficult to achieve accurate, even, and reproducible dyeing results using natural dyes. Seasonal factors highly affect the natural dye composition, making it hard to establish a dyeing recipe that achieves the same color shades among different batches^24^.

The main sources of natural colorants in nature are animals, plants, insects, invertebrates, microbes, and minerals^25^. Regarding those dyes from natural resources, they are derived from various natural sources, including roots, leaves, bark, peels, seeds, fruits, and flowers^26^. Plant waste materials have been investigated as environmentally suitable substitutes for a range of textiles, with special interest on natural fibers, such as cotton and wool^27^. Among them, black tea (*Camellia sinensis*) waste has attracted considerable attention as a sustainable and eco-friendly resource for fiber dyeing, offering additional benefits such as antimicrobial properties^28^. Tannin, which is a major component in tea leaves, acts as a bio-mordant, which lessens the use of heavy metal salts as mordants^29^. Additionally, black tea leaves are rich in various substances, including caffeine, carbohydrates, carotenoids, chlorophyll, lipids, minerals, nucleotides, organic acids, polyphenols, saponins, unsaponifiable chemicals, and volatile compounds^30^. The main pigments in black tea are thearubigins and theaflavins, derived from the fermentation of polyphenols present in tea^31^.

With the aim of enhancing the performance attributes of textile fabrics dyed with natural colorants, researchers have utilized nano-materials for pretreatment, post-treatment, or simultaneous treatment of textiles^32,33^. Nano-materials have a great share in various industrial sectors, including dyeing and finishing of textiles solely or in combination with other reagents and tools ^34,35^. Special emphasis has been undertaken on metals and metal oxide nanoparticles (NPs) in imparting certain desired properties to textile substrates, viz., antimicrobial activity^36^, UV protection^37^, electrical conductivity^38^, and self-cleaning^39^. Metal ions bind to the fibers through coordinate bonds between an electron donor atom in the textile substrate, such as nitrogen and oxygen, and electron-acceptor species, namely the metal ions^40^.

Black tea extract has been utilized in dyeing wool, cotton, and natural silk fibers^41^. However, no research work has been reported regarding the use of black tea extract in dyeing viscose fabrics. Herein, an eco-friendly cost-effective sustainable method for dyeing and finishing viscose fabric was proposed as an alternative method for the polluting conventional dyeing of viscose using synthetic dyes. The feasibility of using black tea waste extract and ZnO NPs in sustainable and eco-friendly agents for the dyeing and functional finishing of viscose fibers was systematically investigated. The effect of various dyeing conditions, including dyeing pH, temperature, and duration, as well as the colorant concentration on the color strength of the dyed fabrics, was studied. Lowering the dyeing temperature was the main target of this investigation to decrease energy and water consumption significantly. This would lessen the carbon and water footprint of the final product compared to the conventional dyeing processes of textile fabrics that are usually carried out near the boil^42^.

## Experimental

### Materials

Plain weave viscose fabrics (135 g/m^2^) were purchased from the Misr Company for Spinning and Weaving, El-Mahalla El-Kubra, Egypt. Loose black tea (El-Arosa brand) was purchased from the local market. Acetic acid and sodium carbonate were provided by El Nasr Pharmaceutical Chemicals Company, Cairo, Egypt. Zinc oxide nanoparticles (ZnO NPs) of particle size ˂ 100 nm and MW 81.39 g/mol were supplied by Sigma-Aldrich, USA. The nonionic detergent Egyptol PLM (based on nonyl phenol ethoxylate) was kindly provided by the Starch and Brewer’s Company, Alexandria, Egypt.

### Methods

#### Preparation of black tea waste (spent tea grounds)

Five liters of distilled water were brought to the boil, and then 100 g of loose black tea was added to the boiling water. Maintaining a material-to-liquid ratio (MLR) at 1:50, the mixture was kept at the boil for 5 min. The mixture was filtered through an appropriate sieve to separate the liquid extract from the solid residues. The resulting tea residue (spent tea grounds) was air-dried away from light at room temperature until a constant weight was achieved, ensuring complete moisture removal. The dried residues were subsequently stored in sealed plastic bags under dry conditions at ambient temperature until use^43^.

#### Extraction of natural colorant

Different masses (1.0, 2.0, 3.0, and 4.0 g) of black tea waste extract (BTWE) were dissolved in 100 mL of boiling distilled water. The mixtures were kept at the boil for 10 min and finally filtered to remove any solid impurities. The extracted colored solution was used directly for dyeing of viscose fabrics^44^.

#### Dyeing of viscose fabric

Before dyeing, viscose fabric was scoured by soaking in a 2 g/L non-ionic detergent aqueous solution for 30 min^45^. The fabrics were then rinsed and air-dried at room temperature. The scoured fabric samples were dyed using an aqueous solution of the black tea waste extract (BTWE), and the MLR was 1:50. The dyeing process was carried out in a water bath (Memmert GmbH Co, Germany) at pH 3, 4, 5, 7, and 9 adjusted by acetic acid or sodium carbonate solution. The dyeing temperature ranged between 45, 60, 75, and 90 °C, and the used colorant concentrations were 1.0, 2.0, 3.0, and 4.0% (w/v). The dyeing duration ranged between 30, 45, 60, 75, and 90 min with continuous shaking (140 rpm). After dyeing, the samples were rinsed thoroughly with tap water and left to dry overnight at ambient conditions. It is worthy to mention that dyeing of viscose fabrics with natural and direct dyes is carried out near neutrality^46^; however the adopted range of reaction conditions was selected to match with the nature of both the textile substrate and the colorant. For instance, lowering the dyeing temperature would allow dyeing of viscose at relatively lower pH values without loss in fiber and strength.

#### Treatment with zinc oxide nanoparticles (ZnO NPs)

*Pre-treatment* Viscose fabric was pre-treated by soaking in a bath containing 0.025 g ZnO NPs dispersed in 50 mL distilled water for 30 min at 90ºC. The sample was then removed from the treatment bath and dried at ambient conditions. The pre-treated fabric was dyed using black tea waste extract at pH 3 and 45ºC for 60 min; the MLR was 1:50. The dyed samples were left to dry overnight at room temperature.

*Simultaneous dyeing and treatment* Adopting the optimum dyeing conditions, dyeing and treatment of viscose fabric were conducted in one bath with continuous shaking in the presence of 0.025 g of ZnO NPs. The fabrics were removed from the bath, thoroughly rinsed with tap water, and left to dry overnight.

*Post-treatment *In this case, viscose fabrics were dyed with the BTWE extract at the assigned optimum dyeing. The dyed samples were then post-treated with 0.025 of ZnO NPs at 90 °C for 30 min. The sample was then rinsed thoroughly with tap water and left to dry at ambient conditions.

### Analysis and testing

#### Color measurement

*Color strength *The color strength (K/S) of the dyed viscose fabrics was determined using a reflectance spectrophotometer equipped with a DP65 standard illuminant and 10° observer angle at a λ_max_ 360 nm using PERKIN–ELMER Lambda 3B UV/V spectrophotometer. The K/S values were calculated according to the *Kubelka Munk* Equation^47^, which relates the reflectance (R) of a sample to its absorption (K) and scattering (S) coefficients as follows:

1 $$Color\;strength \left( {K/S} \right) = \frac{{\left( {1 - R} \right)^{2} }}{2R} - \frac{{\left( {1 - R^{^\circ } } \right)^{2} }}{{2R^{^\circ } }}$$

where K and S are the absorption and scattering coefficients, respectively; “R” is the decimal fraction of the reflectance of the dyed sample, and “R” is the corresponding value of the undyed viscose fabric.

*Colorimetric data *The colorimetric analysis of dyed viscose fabrics was carried out using a Spectra DP 9000 reflectance spectrophotometer under standardized conditions (D65 illuminant and 10° standard observer). The color coordinates were recorded in the framework of the CIELAB color spaces^48^. The L*, a*, b*, and *h* values determine the extent of lightness, redness, yellowness, and hue of the dyed viscose fabrics, respectively.

*Fastness properties of the dyed viscose fabrics *The dyed viscose fabrics’ fastness to the effect of washing, crocking (dry and wet), perspiration, and light was assessed using the respective standard test method.

The colorfastness against washing was evaluated according to the standard test method ISO 105-C06 (2010). The colorfastness to laundering is used to evaluate the ability of the dyed or printed textile substrate to resist the color change as well staining to other textile item of the same substrate during laundering^49^.

The standard method ISO 105-B02 (2014) was adopted to evaluate the colorfastness of the dyed samples to light. In this test, the examined textile specimen is exposed to an artificial light source, along with reference substances. The colorfastness is recorded after comparing the alteration in the color of the tested sample with that of the corresponding reference material^50^.

The colorfastness against crocking (dry and wet) was assessed using the AATCC Technical Manual, Method 8 (1989), 68, 23 (1993). This test was designed to evaluate the resistance of the colored textile substrate to crocking through determination of the extent of transferring the color from the dyed fabric to another surface^51^.

The AATCC test method (Technical Manual, Method 15, 1989) was followed to assign the colorfastness of the dyed viscose fabrics to perspiration. The human body’s perspiration may be either acidic or alkaline. This method assigns the extent of the dyed specimen’s ability to withstand fading under the influence of an artificial perspiration^52^.

#### Antimicrobial properties

The antibacterial activity of the dyed and the undyed fabrics against *Staphylococcus aureus* (ATCC 6538-P), *Escherichia coli* (ATCC 25933), and *Candida albicans* (ATCC 10231) was evaluated using the colony-forming unit (CFU) method. Each microbial strain was inoculated with 10 µL and 10^8^ CFU/mL of nutritious broth (pH 6.8) for bacteria and potato dextrose broth for fungi, respectively. Fabric samples (1 cm^2^/250 mg) were added to the infected media, and they were cultured for 24 h at 37 °C for bacteria and 28 °C for fungi. Growth reduction was calculated by comparing the absorbance decline to untreated controls. The growth reduction was calculated from the following equation:

2 $${\mathrm{R}}\left( {{\% }} \right) = \frac{B - A}{B} \times 100$$

where A is the number of colonies of the culture control sample, while B is the number of colonies of dyed sample.

#### Durability test

The durability of the dyed fabrics after repeated washing was estimated in terms of their microbial resistance in accordance with the AATCC 61-1989 standard method. The dyed sample was washed for up to 20 wash cycles in a launder-o-meter for 45 min at 40 °C using a detergent.

#### Antioxidant assay

The antioxidant activity of any polymeric material is highly important as it determines the rate of fiber deterioration under the influence of UV radiation or by aging^53^. The antioxidant activity of the dyed as well as undyed viscose fabrics was evaluated by adopting the radical cation decolorization assay using 2,2′-azinobis-(3-ethylbenzothiazoline-6-sulphonic acid) (ABTS)^54^. The radical cations of ABTS (ABTS·^+^) were obtained by reacting a 0.00245 M aqueous solution of potassium persulfate with a 0.007 M ABTS solution. The mixture was maintained at 30 °C for about 12 h in a dark place. Afterwards, the solution was diluted with a phosphate buffer (0.1 M, pH 7.4). The test specimen (*ca.* 10 mg fabric) was steeped in 10 mL of ABTS·^+^ solution for half an hour. Finally, the antioxidant activity was calculated at 734 nm using Eq. 3:

3 $${\mathrm{Antioxidant}}\;{\mathrm{activity}}\left( \% \right) = \left[ {{{\left( {{\mathrm{A}}_{{1}} {-}{\mathrm{A}}_{{2}} } \right)} \mathord{\left/ {\vphantom {{\left( {{\mathrm{A}}_{{1}} {-}{\mathrm{A}}_{{2}} } \right)} {{\mathrm{A}}_{{1}} }}} \right. \kern-0pt} {{\mathrm{A}}_{{1}} }}} \right] \times {1}00$$

where A_1_and A_2_ are the ABTS·^+^ absorbance before impregnation of the fabric and that of the remaining ABTS·^+^ after impregnation with the fabric, respectively. Every recorded value is an average of three parallel tests.

#### Fourier-transform infrared spectroscopy (FTIR)

The FTIR spectra of the untreated PA fabric as well as that dyed with BTWE were inspected using the FTIR spectrophotometer (Nexus 670; Nicolet, United States) in the transmission mode. The data was recorded within the range of 4000–400 cm^-1^ and the spectral resolution of 4.0 cm^−1^.

#### Scanning electron microscopy

A scanning electron microscopic study was carried out on the undyed as well as the dyed viscose fabrics in the presence and absence of mordant using a 30 kV scanning electron microscope, JEOL (Japan) Model JSM-6360.

#### Fabric properties

By following the AATCC standard test method 183:2010-UVA Transmittance, the ultraviolet protection factors (UPF) of the dyed as well as undyed viscose fabrics were evaluated on a UV spectrophotometer (Shimadzu 3101 Spectrophotometer, Kyoto, Japan). The ASTM D 76 standard method was adopted to determine the tensile strength and elongation at the break of the test specimens using the Instron Textile Tester (USA). Every presented result is the main value of three trials.

#### Statistical analysis

The relationship between the different dyeing process parameters, namely pH, dye concentration, dyeing temperature and duration, and the K/S, as well as the colorimetric data of the dyed fabrics, was tested using the one-way ANOVA test.

All reported color strength and colorimetric data of the dyed samples, as well as their UPF and tensile properties, were the average of at least three measurements. The standard deviation for each value was reported in the respective table in the results and discussion section. On the other side, an error bar was shown in the results of the antimicrobial properties of the dyed fabric after repeated washing, as shown in the corresponding figures in the results and discussion section.

## Results and discussion

The dyeability of a textile substrate depends on many factors, such as the chemical structure of the dye as well as the substrate, the dye concentration, the dyeing temperature and duration, and the pH of the dyeing bath^55^. The pre-treatment and finishing process carried out on the textile substrate before and after dyeing may also affect the color strength as well as other colorimetric data of the dyed fabric^56^.

### Effect of dye-bath pH

The pH of the dyeing bath is of prime importance in determining the color strength (K/S), chromatic values, and the colorfastness of the dyed fibers^57^. The effect of dyeing bath pH on the color strength (K/S) and colorimetric data of the viscose fabrics dyed with BTWE was investigated, and the results were shown in Table 1. The data in this table revealed that the K/S of the dyed fabrics increased significantly as the dye-bath pH decreased from slightly to strongly acidic conditions. A maximum K/S value (7.53) was attained at pH 3. In neutral and slightly alkaline media, the color strength decreased remarkably to 2.02 and 1.80, respectively. A darker fabric was also produced in acidic media, as indicated by the diminishing lightness (L*) values. The data in this table also indicated that as the pH increased, the redness-greenness coordinate (a*) decreased, implying a shift toward redder tones. A similar trend with a relatively higher rate of decrement was encountered for the yellowness-blueness coordinate (b*), demonstrating stronger yellow tones. These results confirmed that the K/S and colorimetric data of the viscose fabrics dyed with BTWE have been strongly sensitive towards the pH of the dyeing bath.

**Table 1:** Effect of the dyebath pH on the color strength (K/S), Lightness (L*), Redness-Greenness (a*) and Yellowness-Blueness (*b)* of viscose fabrics dyed with BTWE (dyeing conditions: 2% BTWE for 60 min at 90 °C; MLR 1:50) [Each reported value is an average of at least three measurements**].**

Dye-bath pH	K/S	L*	a*	b*
3	7.53 ± 0.02	64.70 ± 0.02	5.04 ± 0.02	20.18 ± 0.01
4	3.61 ± 0.01	71.79 ± 0.02	3.55 ± 0.03	9.52 ± 0.02
5	3.02 ± 0.01	72.63 ± 0.01	3.29 ± 0.02	8.00 ± 0.03
7	2.02 ± 0.02	74.96 ± 0.02	2.62 ± 0.02	5.66 ± 0.03
9	1.80 ± 0.03	79.08 ± 0.02	2.55 ± 0.03	− 1.64 ± 0.03

The increased dye uptake in highly acidic conditions may be due to the lower degree of ionization of the hydroxyl and carboxylic groups in the chemical structure of most of the colored compounds in the BTWE. This would noticeably decrease the negative charge on the surface of the fibers, thus minimizing electrostatic repulsion with the –OH groups in viscose fabrics, allowing the formation of hydrogen bonds between the dye molecules and the fibers’ macromolecular chains. It has been concluded that acidic media promote stronger hydrogen bonds and ion-dipole interactions between the dye molecules and the textile substrates. Accordingly, the hydroxyl groups of viscose can form hydrogen bonds with the corresponding groups in polyphenols found in BTWE, resulting in improved dye uptake^58^.

**Fig. 1:**
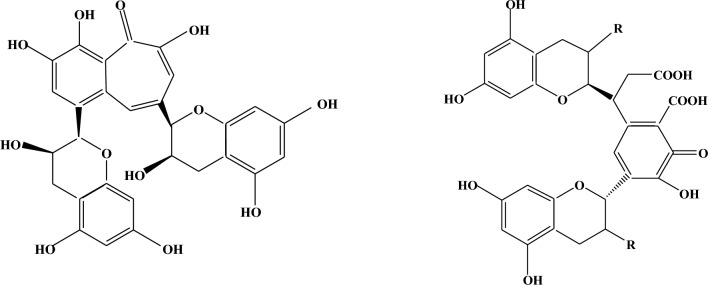
The structural formulae of theaflavin (left) and thearubigin (right).

The most abundant secondary polyphenol pigments in BTWE that imparted its characteristic color are theaflavins, thearubigins, theasinensins, theacitrins, and oolongtheanins^31,59^. The chemical structures of all theaflavins, namely theaflavin, theaflavin-3-gallate, theaflavin-3′-gallate, and theaflavin-3,3′-digallate, are based on benzotropolone (a seven-membered ring ketone) fused with two phenolic structures. Under the acidic dyeing conditions for viscose fabric with BTWE, theaflavins are stable, containing a number of –OH groups that can form hydrogen bonds with the hydroxyl groups of any polysaccharide substrates, such as cotton and viscose^60^. The carboxylic and hydroxyl groups in the reddish-brown polyphenol “thearubigins” are suitable candidates for similar hydrogen bonding with viscose fabric at pH less than seven^61^. The structural formulae of theaflavin and thearubegin are shown in Fig. 1.

### Effect of dyeing temperature

The K/S of viscose fabrics dyed with BTWE was found to be highly dependent on the dyeing temperature. The results in Table 2 demonstrated that the dye uptake reached a maximum K/S value of 7.53 at 90 °C. The least lightness (L*) value of 64.70 was obtained at the same temperature, which supported this finding. On the other hand, the K/S value was least at 75 °C, indicating a lighter color with lower color absorption. Remarkably, at 45 °C, the greatest a* (redness) and b* (yellowness) values were observed, proposing a warmer tone at lower temperatures.

**Table 2:** Effect of dyeing temperature on the K/S, Lightness (L*), Redness-Greenness (a*) and Yellowness-Blueness (b*) of viscose fabrics dyed with BTWE (dyeing conditions: 2% BTWE for 60 min at pH 3; MLR 1:50) [Each reported value is an average of at least three measurements].

Dyeing temperature (°C)	K/S	L*	a*	b*
45	6.68 ± 0.02	69.02 ± 0.02	6.08 ± 0.03	22.02 ± 0.02
60	5.23 ± 0.01	72.78 ± 0.03	5.11 ± 0.02	20.93 ± 0.03
75	4.55 ± 0.01	77.85 ± 0.03	5.08 ± 0.02	20.42 ± 0.03
90	7.53 ± 0.01	64.70 ± 0.03	5.04 ± 0.02	20.18 ± 0.03

These findings may be attributed to the heat-induced disruption of intramolecular hydrogen bonds within the vicinity of viscose fabrics, which loosened the fiber structure, enhanced swelling, and allowed dye molecules to penetrate deeply into the matrix of fibers^58^. However, high temperatures might cause partial degradation of bioactive compounds, especially flavonoids, thereby lowering the fabric’s functional characteristics, such as antioxidant or antibacterial activity^62^. At these temperatures (60 °C and 75 °C), partial thermal degradation of flavonoids may happen, coupled with insufficient fiber swelling. This lowers dye molecules’ effective penetration into the viscose matrix, decreasing color strength. In contrast, dyeing at 45 °C led to the best balance between two counteracting actions. The temperature was high enough to permit the dye to penetrate the fibers without destroying the BTWE’s sensitive natural components. As a result, 45 °C could be the appropriate dyeing temperature since it gave good color strength, preserved the fabric’s functional characteristics, and, most important, saved energy. The results of this study are consistent with previous investigations that assigned the ideal dyeing temperatures for flavonoids and phenols at 45–50 °C and 50–55 °C, respectively^63^. Similarly, flavonoids are better preserved when dyed at moderate temperatures (45 to 50 °C), which prevents the compounds from degrading and maintains the fabric’s functional characteristics.

### Effect of BTWE concentration

The color intensity of the dyed viscose fabric would be influenced by the concentration of the used BTWE involved in the dyeing process. The data in Table 3 revealed how the K/S increased as the dye concentration increased from 1 to 4%. The greatest value (7.00) was achieved at 4%, whereas the minimum K/S (6.04) was recorded upon dyeing of viscose fabric with an aqueous solution containing 1% BTWE. The K/S values increased by only 15.9% when the concentration of the dye solution increased fourfold, indicating that 1% of the colorant has enough functional groups that are able to bind with most of the available functional groups along the macromolecular chains of viscose fabrics. Consequently, the increase in the K/S values is not so high upon increasing the dye concentration. As concentration increased, the lightness (L*) values got lower, proposing that the fabric became darker. Yellowness (b*) increased from 20.60 to 26.47, and redness (a*) increased from 5.05 at 1% to 6.09 at 4%. This shows that as the dye concentration increased, the fabric developed a warmer tone, appearing more red and yellow.

**Table 3:** Effect of dye concentration on the (K/S), Lightness (L*), Redness-Greenness (a*) and Yellowness-Blueness (b*) of viscose fabrics dyed with BTWE (Dyeing conditions: 60 min at 45 °C and pH 3; MLR 1:50) [Each reported value is an average of at least three measurements].

Dye bath conc. (% w/v)	K/S	L*	a*	b*
1%	6.04 ± 0.02	71.38 ± 0.03	5.05 ± 0.02	20.60 ± 0.02
2%	6.68 ± 0.03	77.85 ± 0.03	6.08 ± 0.03	22.02 ± 0.02
3%	6.78 ± 0.02	69.75 ± 0.02	6.03 ± 0.03	24.20 ± 0.03
4%	7.00 ± 0.02	64.38 ± 0.01	6.09 ± 0.01	26.47 ± 0.02

### Effect of dyeing time

The dyeing period certainly impacts how much color the viscose fabric absorbed from the BTWE. As indicated in Table 4, coloring the fabric for a longer period of time improved the color strength (K/S), which reached its highest point (7.00) at 60 min. This means that the fabric absorbed the most dye during this time. After 60 min, the K/S value began to diminish slightly. It seems that after 60 min, the dyeing operation reached an equilibrium state between the diffusion of dye molecules from the dye bath towards the fiber’s interior and desorption of the dye from the fiber’s vicinity into the dye solution. A dyeing time of 60 min can be regarded as appropriate for accomplishing most extreme color strength under the conditions specified. This assumption is in harmony with previous finding, which demonstrated that a greater K/S value results from an increase in dye concentration as long as the fabric can absorb more dye^64^. Besides, extending the dyeing time increased color strength until a certain point, at which point the dye reached equilibrium or diffused back into the dye bath, causing the K/S value to begin declining. The data in Table 4 also indicated that the dyed fabrics became darker at 60 min. As the lightness (L*) value diminished, the redness (a*) and yellowness (b*) were both at their highest point, showing a stronger and warmer color.

**Table 4:** Effect of dyeing time on the color strength (K/S), Lightness (L*), Redness-Greenness (a*), Yellowness-Blueness (b*), of viscose fabrics dyed with BTWE (Dyeing conditions: 4% BTWE at 45 °C pH 3; MLR 1:50) [Each reported value is an average of at least three measurements].

Dyeing time (min)	K/S	L*	a*	b*
30	4.04 ± 0.01	74.45 ± 0.02	4.01 ± 0.02	7.06 ± 0.03
45	4.87 ± 0.01	72.94 ± 0.02	4.22 ± 0.02	19.30 ± 0.02
60	7.00 ± 0.02	64.38 ± 0.01	6.09 ± 0.01	26.47 ± 0.02
75	4.89 ± 0.01	71.80 ± 0.02	4.19 ± 0.03	18.51 ± 0.03
90	5.33 ± 0.02	72.85 ± 0.02	4.07 ± 0.02	17.08 ± 0.02

The assigned optimum dyeing conditions of viscose fabric with BTWE were found to be economically feasible and industrially applicable. The pH was adjusted at 3 using a relatively cheap reagent (commercial acetic acid) at 45ºC leading to lowering energy and water consumption significantly, and thus decreasing the carbon and water footprint of the final product. Commercial dyeing of textile fabrics are usually conducted at 90ºC or higher^65^. These findings rendered BTWE a sustainable and eco-friendly candidate for dyeing cellulosic substrates.

### Effect of zinc oxide nanoparticles (ZnO NPs)

The influence of treating viscose fabric with ZnO NPs on the color characteristics of viscose fabrics dyed with BTWE was monitored. Viscose fabrics were treated with ZnO NPs before, during, and after the dyeing process. As shown in Table 5, simultaneous dyeing and finishing of viscose fabrics with ZnO NPs led to the maximum K/S (9.70) of the dyed fabric, indicating greater dye uptake and a darker shade. The same sample exhibited the lowest lightness (L*) value, resulting in a darker fabric appearance, as well as higher a* and b* values, showing improved reddish and yellowish tones. On the other hand, the pre-treatment of viscose fabric with ZnO NPs before dyeing resulted in a limited increase in the K/S of the dyed sample. In contrast; post-treatment of the dyed viscose sample with ZnO NPs caused a sharp decrease in the K/S of the dyed fabric.

**Table 5:** Color strength (K/S), Lightness (L*), Redness-Greenness (a*) and Yellowness-Blueness (b*) of viscose fabrics dyed with BTWE in the presence of ZnO NPs (Dyeing conditions: 4% BTWE, pH 3, 45 °C, 60 min; MLR 1:50) [Each reported value is an average of at least three measurements**].**

Treatment Methods	K/S	L*	a*	b*
Dyeing only	7.00 ± 0.02	72.38 ± 0.01	4.11 ± 0.01	19.47 ± 0.02
Pre-treatment	7.23 ± 0.03	67.04 ± 0.03	6.00 ± 0.02	22.65 ± 0.03
Meta-treatment	9.70 ± 0.04	64.47	6.48 ± 0.03	21.91 ± 0.03
Post-treatment	3.63 ± 0.02	69.90	3.23 ± 0.03	9.17 ± 0.02

Simultaneous dyeing with BTWE and finishing using ZnO NPs of viscose fabrics resulted in the maximum K/S of the dyed fabric, which might be due to enhancement of the bonding between the dye molecules, ZnO NPs, and fiber, most likely due to changes in fiber surface energy and dye stabilization within the bath^66^. The observed color strength can also be due to the ZnO nanoparticles’ unique capability in interactions with –OH groups from tea polyphenols. It has been agreed that polyphenolics, especially flavonoids, are suitable candidates for chelating heavy metal ions such as Zn^+2^ and Cd^+2^ ions^67^. This would increase their reactivity and allow for stronger attachment to the fiber surface, resulting in better dye fixing and deeper, more distinctive coloration^68^. Post-treatment of the dyed viscose fabrics with ZnO NPs, however, had a negative impact on the K/S of the dyed fabric, presumably because the ZnO interacted exclusively with the exterior layer of already-fixed dye, resulting in dye migration from the fibers’ surface to the dye solution.

**Fig. 2:**
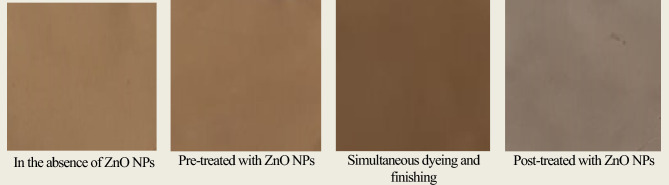
Light photographs of viscose fabrics dyed with BTWE in the presence and absence of ZnO NPs.

Figure 2 showed the light photographs of viscose fabrics dyed with BTWE in the presence and absence of ZnO NPs. The visual discrepancies among the dyed samples are consistent with the K/S values reported in Table 5.

### Statistical analysis

An ANOVA test and Pearson correlation were adopted to assess the significance of the correlation between the dyeing parameters and the color strength as well as the colorimetric data of the dyed viscose fabrics. The *p*-values ≤ 0.05 indicate a significant correlation, and those ≤ 0.01 mean a highly significant correlation. Pearson correlation (r) shows the strength and direction of the relationship between the correlated items. Values close to “+ 1” imply a strong direct relationship, values close to “− 1” indicate a strong inverse relationship, and values close to “0” mean that there is a weak or no relationship.

Table 6 shows the relation (if any) between the pH of the dyeing bath and the color strength and colorimetric data of the dyed fabric. The results in this table indicated that the pH and K/S are strongly correlated, as indicated by the *p*-value (0.005), in an inverse relationship as indicated by the “r” value of − 0.903. A similar trend was found regarding the relation between the dyeing pH and a* and b* values. On the contrary, the values of L* of the dyed fabrics are negatively affected and highly dependent on the pH of the dyeing bath. The data of Table 6 demonstrated also that as the color of the dyed sample got deeper, as indicated by higher K/S values, the L* value got lower.

**Table 6:** Correlation between the dyeing pH and the K/S and colorimetric data of the dyed viscose fabrics.

		pH	K/S	L*	a*	b*
pH	Pearson correlation (r)	3	0.903	0.927	0.961	0.957
	*p*-value		0.005	0.003	0.001	0.001
	N	7	7	7	7	7
K/S	Pearson correlation (r)	0.903	1	0.924	0.955	0.965
	*p*-value	0.005		0.003	0.001	0.000
	N	7	7	7	7	7
L*	Pearson correlation (r)	0.927	0.924	1	− 0.928	0.921
	*p*-value	0.003	0.003		0.003	0.003
	N	7	7	7	7	7
a*	Pearson correlation (r)	0.961	0.955	0.928	1	0.964
	*p*-value	0.001	0.001	0.003		0.000
	N	7	7	7	7	7
b*	Pearson correlation (r)	− 0.957	0.965	− 0.921	0.964	1
	*p*-value	0.001	0.000	0.003	0.000	
	N	7	7	7	7	7

The results declared in Table 7 showed that the dyeing temperature has a very strong negative effect on lightness (L*) (r = − 0.998, *p* = 0.002), indicating that darker shades were obtained when the temperature was raised. There is also a significant negative correlation between temperature and b* (r = − 0.953, *p* = 0.047), indicating that higher temperatures reduce the yellow tone of the fabric. However, the effect of temperature on K/S (r = 0.178, *p* = 0.822) and a* (r = − 0.809, *p* = 0.191) was not significant, meaning that in our investigation, raising the temperature has no strong effect on the K/S of the dyed samples. Finally, L* and b* are strongly positively correlated (r = 0.965, *p* = 0.035), meaning lighter fabrics tend to have higher yellowness.

**Table 7:** Correlation between the dyeing temperature and the K/S, L*, a*, and b* values of the dyed fabrics.

		Temp	K/S	L*	a*	b*
Temp	Pearson correlation (r)	1	0.178	− 0.998	− 0.809	− 0.953
	*p*-value		0.822	0.002	0.191	0.047
	N	4	4	4	4	4
K/S	Pearson correlation (r)	0.178	1	− 0.153	0.293	0.100
	*p*-vale	0.822		0.847	0.707	0.900
	N	4	4	4	4	4
L*	Pearson correlation (r)	− 0.998	− 0.153	1	0.839	0.965
	*p*-value	0.002	0.847		0.161	0.035
	N	4	4	4	4	4
a*	Pearson correlation (r)	− 0.809	0.293	0.839	1	0.943
	*p*-value	0.191	0.707	0.161		0.057
	N	4	4	4	4	4
b*	Pearson correlation (r)	− 0.953	0.100	0.965	0.943	1
	*p*-value	0.047	0.900	0.035	0.057	
	N	4	4	4	4	4

Table 8 shows the correlation between the dye concentration and the K/S, L*, a*, and b* values of the dyed viscose fabrics. The data indicated that there is a very strong negative correlation between dye concentration and L* (r = − 0.998, *p* = 0.002). When the dye concentration increases, the L* value decreases, meaning the fabric becomes darker. Similarly, there is a strong negative correlation between the dye concentration and b* (r = − 0.953, *p* = 0.047). Higher dye concentration leads to less yellowness. On the other hand, the relationship between the dye concentration and a* (r = − 0.809, *p* = 0.191) is not significant, meaning dye concentration does not strongly affect redness. The relationship between concentration and K/S of the dyed samples (r = 0.178, *p* = 0.822) is very weak and not significant, meaning K/S is not strongly affected by dye concentration in this range. A strong positive correlation exists between L* and b* (r = 0.965, *p* = 0.035). Fabrics with higher L* (lighter shades) also have higher b* values (more yellow tones).

**Table 8:** Correlation between the dye concentration and the K/S, L*, a*, and b* values of the dyed fabrics.

		Dye conc.	K/S	L*	a*	b*
Dye conc.	Pearson correlation (r)	1	0.178	− 0.998	− 0.809	− 0.953
	*p*-value		0.822	0.002	0.191	0.047
	N	4	4	4	4	4
K/S	Pearson correlation (r)	0.178	1	− 0.153	0.293	0.100
	*p*-value	0.822		0.847	0.707	0.900
	N	4	4	4	4	4
L	Pearson correlation (r)	− 0.998	− 0.153	1	0.839	0.965
	*p*-value	0.002	0.847		0.161	0.035
	N	4	4	4	4	4
a	Pearson correlation (r)	− 0.809	0.293	0.839	1	0.943
	*p*-value	0.191	0.707	0.161		0.057
	N	4	4	4	4	4
b	Pearson correlation (r)	− 0.953	0.100	0.965	0.943	1
	*p*-value	0.047	0.900	0.035	0.057	
	N	4	4	4	4	4

On the other side, the data in Table 9 implied that the dyeing time had no significant effect on K/S and color parameters. Nevertheless, a strong negative correlation was observed between K/S and L*, confirming that higher color strength results in darker shades. Moreover, K/S was positively correlated with a*, and a* showed a strong positive correlation with b*, indicating that increased redness is accompanied by higher yellowness, producing warmer color tones.

**Table 9:** Correlation between the dyeing time and the K/S, L*, a*, and b* values of the dyed fabrics.

		Time	K/S	L*	a*	b*
Time	Pearson correlation (r)	1	0.375	− 0.173	− 0.314	0.492
	*p*-value		0.534	0.781	0.686	0.400
	N	5	5	5	4	5
K/S	Pearson correlation (r)	0.375	1	− 0.953	0.961	0.864
	*p*-value	0.534		0.012	0.039	0.059
	N	5	5	5	4	5
L*	Pearson correlation (r)	− 0.173	− 0.953	1	− 0.992	− 0.785
	*p*-value	0.781	0.012		0.008	0.115
	N	5	5	5	4	5
a*	Pearson correlation (r)	− 0.314	0.961	− 0.992	1	0.988
	*p*-value	0.686	0.039	0.008		0.012
	N	4	4	4	4	4
b*	Pearson correlation (r)	0.492	0.864	− 0.785	0.988	1
	*p*-value	0.400	0.059	0.115	0.012	
	N	5	5	5	4	5

### Fastness properties

Evaluating the colorfastness of textile fabrics dyed or printed with natural colorants against washing, crocking, perspiration, and light is of prime importance to ensure the process feasibility^69^. The colorfastness of the undyed as well as dyed viscose fabrics with BTWE in the presence and absence of ZnO NPs was investigated, and the results were summarized in Table 10. The data in this table elucidated that most of the examined viscose samples had good to very good fastness against washing, perspiration, crocking, and light. Samples dyed with viscose BTWE in the presence of ZnO NPs had excellent fastness against crocking. The colorfastness against washing rating for both color alteration and staining varied between 3 and 4, demonstrating a satisfactory resistance to washing^70^. The light fastness was reliably rated as 6 on the gray scale, which showed extremely good resistance with just minor fading. Similar ratings were observed for the colorfastness against perspiration regarding the staining if washed in the presence of cotton, wool, and polyester fabrics. Significantly, at the optimum dyeing conditions, simultaneous dyeing and treatment with ZnO NPs outperformed the pretreatment with the NPs followed by dyeing in terms of acidic sweat fastness. This indicated that adding ZnO NPs during dyeing of viscose with BTWE resulted in stronger dye fixing^71^.

**Table 10:** Fastness properties of viscose fabrics dyed with BTWE in the presence and absence of ZnO NPs (dyeing conditions: pH 3, 60 min, 45 °C; MLR. 1:50).

Dyed viscose fabrics	Washing				Perspiration								Crocking		Light
	Alt	St			Acidic				Alkaline				Dry	Wet	
		C	W	PE	Alt	St			Alt	St					
						C	W	PE		C	W	PE			
Dyed only	3–4	3–4	3–4	4	4–5	4	4	4	3–4	4	4	4	4	4	6
Pre-treated with ZnO NPs then dyed	3–4	3–4	3–4	4	4–5	4	4	4	3–4	4	4	4	5	5	6
Dyed in the presence of ZnO NPs	3–4	3–4	3–4	4	5	5	5	5	3–4	5	5	5	5	5	6

Alt, Alteration ;St, Staining; C, Cotton fabric; W, Wool fabric; PE, Polyester fabric.

### Antimicrobial activity

One of the major disadvantages of natural fibers, as well as those regenerated from cellulosic sources, such as viscose fibers, is their attack by microorganisms during wear and storage^72^. Accordingly, it was essential to evaluate the antimicrobial activity of dyed viscose fabric against Gram-positive (*Staphylococcus aureus*), Gram-negative (*Escherichia coli*), and fungus (*Candida albicans*). As shown in Table 11, the undyed fabrics had almost no resistance to the three species used in this investigation. Dyeing of viscose with BTWE rendered the fabric remarkable microbial resistance to different extents, with reduction percent in the order *E. coli* (78.07%) < *C. albicans* (85.82%) < *S. aureus* (91.67%). Viscose fabrics dyed with BTWE in the presence of ZnO NPs exhibited excellent microbial resistance towards the three examined species in the order: *E. coli* (98.88%) > *C. albicans* (96.27%) ≈ *S. aureus* (96.67%).

**Table 11:** Antimicrobial activity of undyed viscose as well as dyed fabrics with BTWE in the presence and absence of ZnO NPs against *Staphylococcus aureus, Escherichia coli, and Candida albicans.*

Viscose Fabric	Colony forming units (CFU) (× 10^3^)	
	No. of colonies	Reduction (R%)
Against 150 *S. aureus* Colonies		
Untreated and undyed	145	3.33
Dyed only	13	91.67
Dyed in the presence of ZnO NPs	5	96.67
Against 269 *E. coli* Colonies		
Untreated and undyed	372	0.00
Dyed only	59	78.07
Dyed in the presence of ZnO NPs	3	98.88
Against 423 *C. albicans* Colonies		
Untreated and undyed	465	0.00
Dyed only	110	85.82
Dyed in the presence of ZnO NPs	598	96.27

**Fig. 3:**
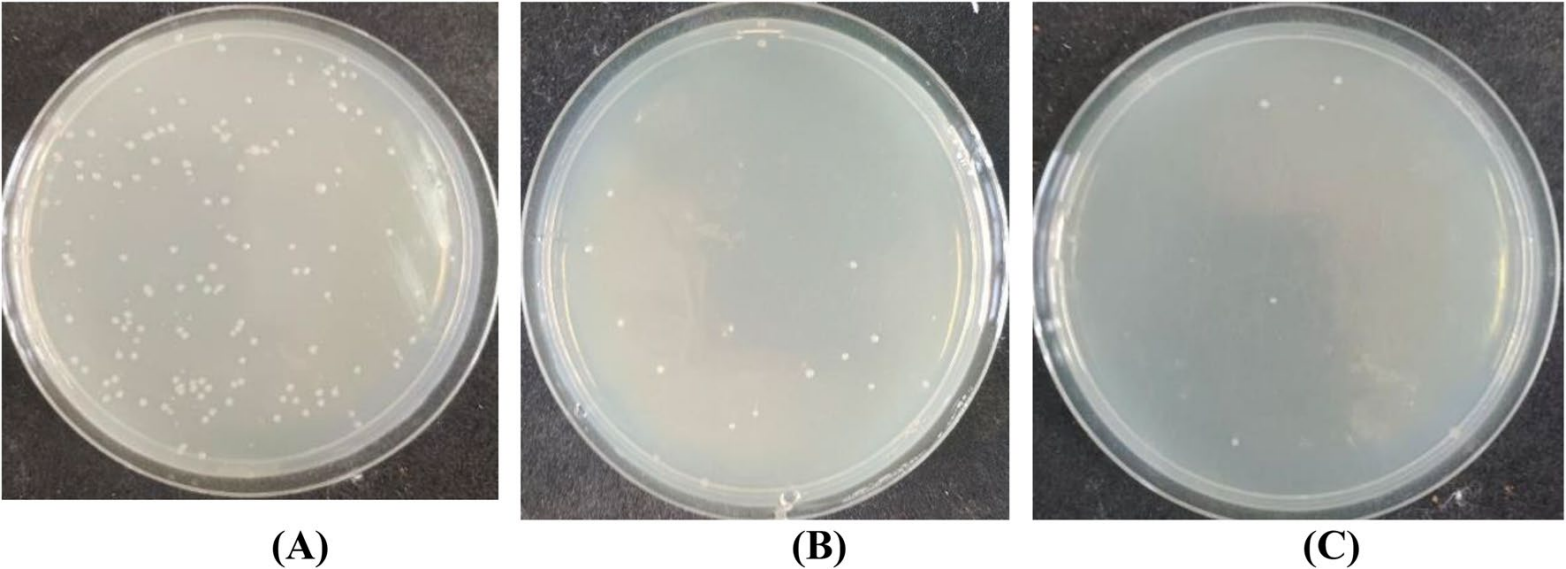
Antibacterial activity of viscose fabrics against *S. aureus*. (**A**) undyed, (**B**) dyed with BTWE, and (**C**) dyed with BTWE in the presence of ZnO NPs.

**Fig. 4:**
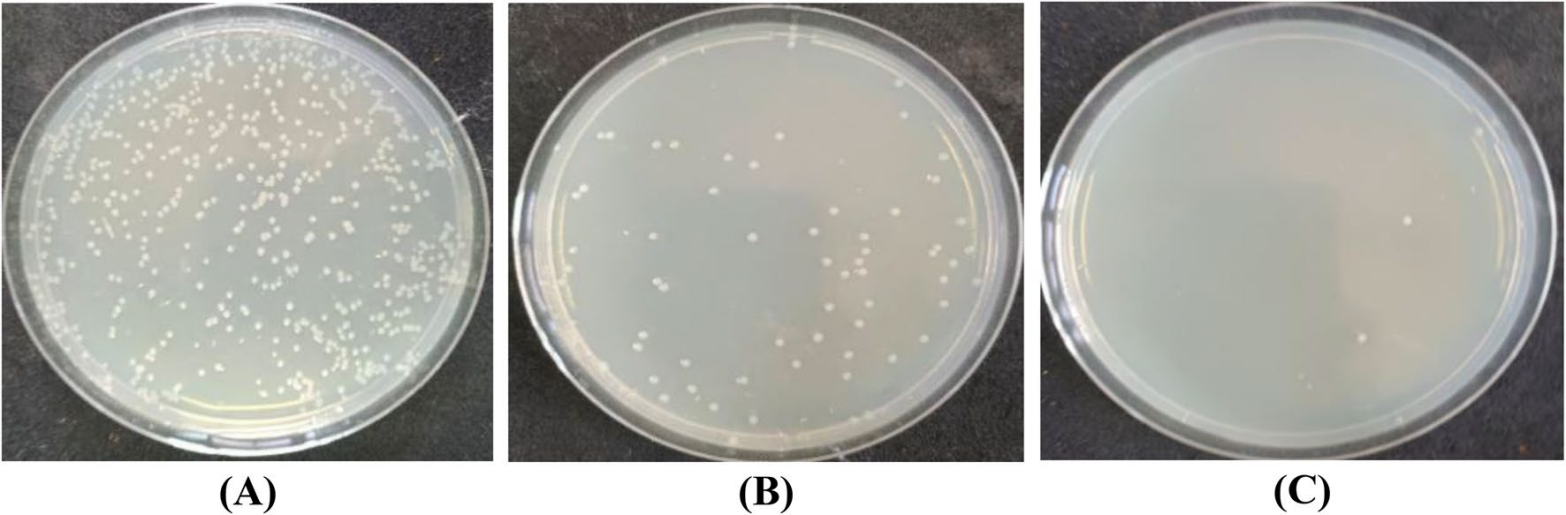
Antibacterial activity of viscose fabrics against *E. coli*. (**A**) undyed, (**B**) dyed with BTWE, and (**C**) dyed with BTWE in the presence of ZnO NPs.

**Fig. 5:**
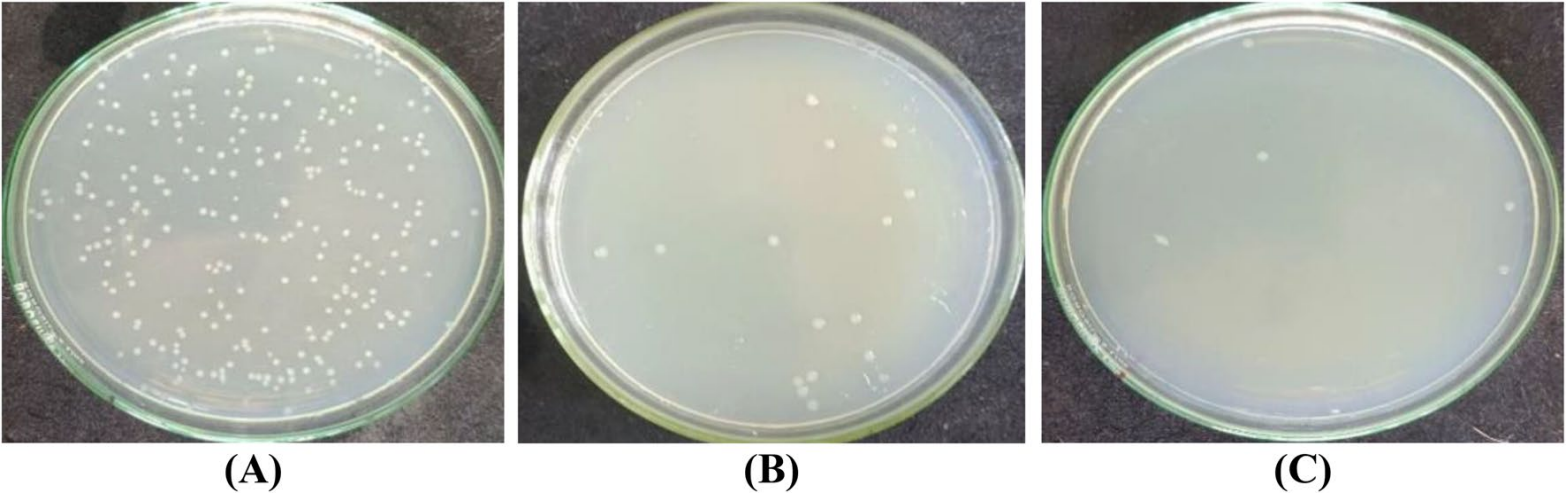
Antifungal activity of viscose fabrics against *C. albicans*. (**A**) undyed, (**B**) dyed with BTWE, and (**C**) dyed with BTWE in the presence of ZnO NPs.

These results confirmed the synergistic effect of ZnO NPs inherent antibacterial properties, and that of the polyphenolic compounds in the black tea extract (BTWE), which led to enhancing the bactericidal and fungicidal activities of viscose fabrics. It has been agreed that ZnO NPs are capable of disrupting the cell membranes of bacteria and fungi, probably by the production of reactive oxygen species, such as superoxide anion, hydroxyl radicals, and hydroxyl ion^73^. Dyeing of viscose fabric with BTWE in the absence of ZnO NPs also revealed considerable, albeit lower, reductions in microbial growth, presumably due to the presence of polyphenolic compounds in the BTWE whose antimicrobial activity stems from their capacity to create hydrogen bonds with microbial membrane proteins, which in turn causes protein inactivation and metabolic disruption^74^. Tannins, found predominantly in black tea, are known to interfere with oxidative phosphorylation and prevent microbe adhesion, which further suppresses the growth of microorganisms^75^. Figures 3, 4, and 5 showed the plates used for the antimicrobial testing of viscose fabrics against *S. aureus*, *E. coli*, and *C. albicans*, respectively.

**Fig. 6:**
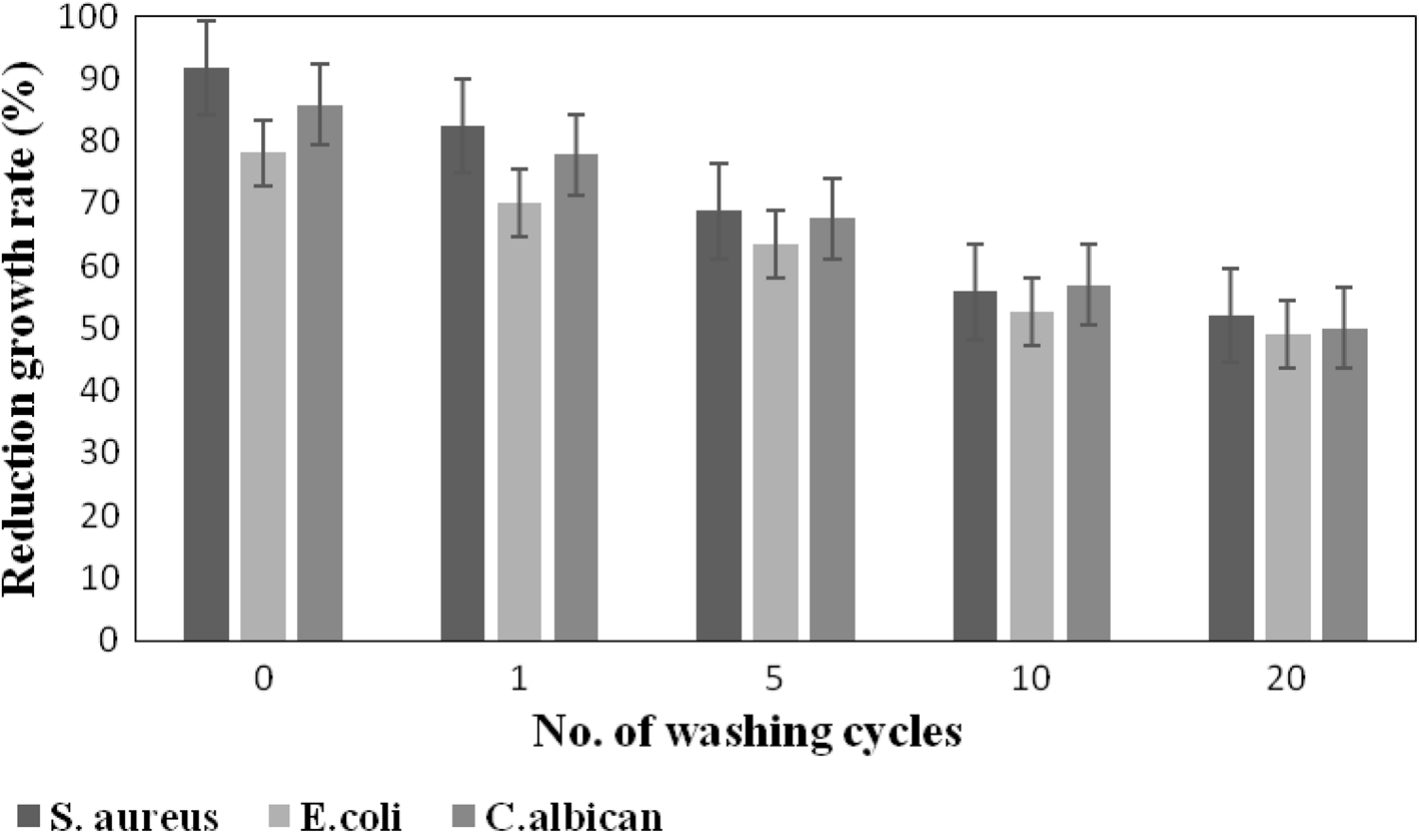
Durability of viscose fabrics dyed with BTWE against *S. aureus*, *E. coli*, and *C. albican.*

**Fig. 7:**
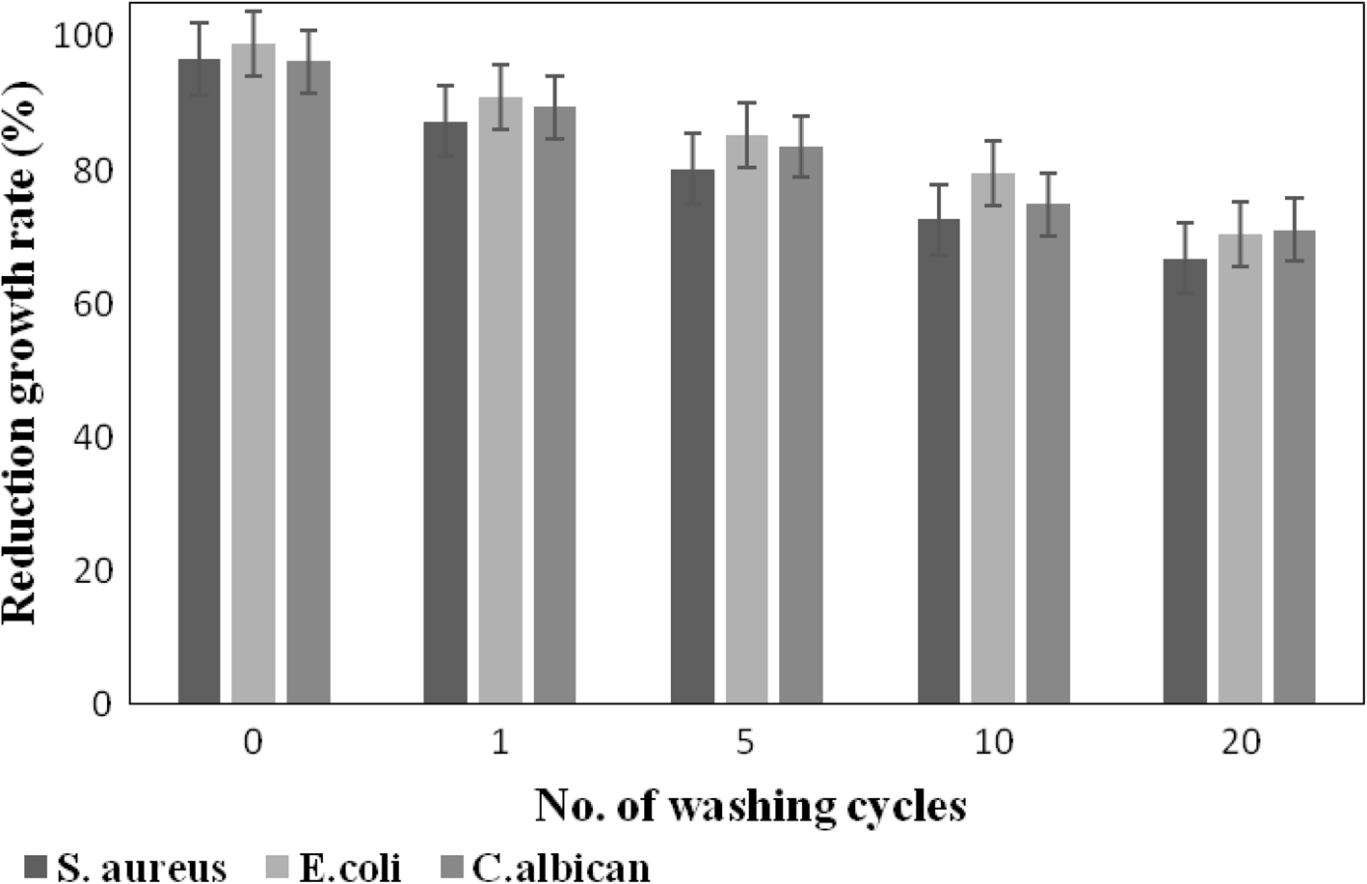
Durability of viscose fabrics dyed with BTWE/ZnO NPs against *S. aureus*, *E. coli*, and *C. albican.*

The durability of the microbial resistance of the finished textile substrates is of prime importance to assign its proper utilization in the medical field^76^. The resistance of the dyed viscose fabric in the presence and absence of ZnO NPs to bacterial and fungal attack was examined after 1, 5, 10 and 20 wash cycles. Figures 6 and 7 prove that the dyeing of viscose fabric with BTWE in the presence of ZnO NPs has better durability up to 20 washing cycles. Hence, the treatment can resist the washing conditions inside washing machines. Thorough inspection of these figures elucidated that the extent reduction in growth rate of viscose fabrics dyed with BTWE/ZnO NPs after 20 wash cycles decreased by 30.9, 28.7, and 22.2% in the case of *S. aureus*, *E. coli*, and *C. albican*, respectively. The corresponding values in the case of viscose fabrics dyed with BTWE without ZnO are 43.2, 37.3, and 33.6%. These findings indicate that the applied reagents were not strongly bonded to the dyed viscose fabric, and the mode of boding between the colorant and textile substrate is physical bonding. Accordingly, the dyed fabrics would be suitable candidate for the fabrication of disposable textiles or in the manufacture of tapestry.

### Antioxidant activity

The dyed as well as undyed viscose fabrics’ antioxidant activity was evaluated using the percentage inhibition and antioxidant capacity, which were expressed as mg of Trolox equivalents per gram sample and per square centimeter of fabric, respectively. The results in this investigation, presented in Table 12, indicated that there is a minimal antioxidant activity in the undyed viscose fabric, with a limited antioxidant capacity and very little inhibiting of free radicals. The antioxidant activity of viscose fabric dyed with BTWE, on the other hand, was significantly increased. This may be attributed to the antioxidant activity of polyphenolic compounds existing in the BTWE, which can form hydrogen bonds with the aliphatic hydroxyl groups along the macromolecular chains of viscose fabrics.

**Table 12:** Antioxidant activity of viscose fabrics dyed with BTWE.

Sample	% inhibition	Antioxidant activity	
		(mg TE/g sample)	(mg TE/cm^2^ sample)
Undyed viscose fabric	0.06	8.283	0.069
Dyed viscose fabric	74.59	924.314	9.428

This hypothesis is supported by the findings of a previous study, which concluded that dyeing textile fabrics with definite natural plant extracts, such as *Azolla pinnata*, increased the antioxidant activity of the dyed fabrics because of their high phenolic and flavonoid contents^26^. By virtue of the induced antioxidant activity, the treated fabrics can withstand the deteriorative action of UV radiation and oxidizing agents, like chlorine, which exist in tap water and swimming pools that may come in contact with viscose garments during daily lives.

### Fabric chemistry and morphology

**Fig. 8:**
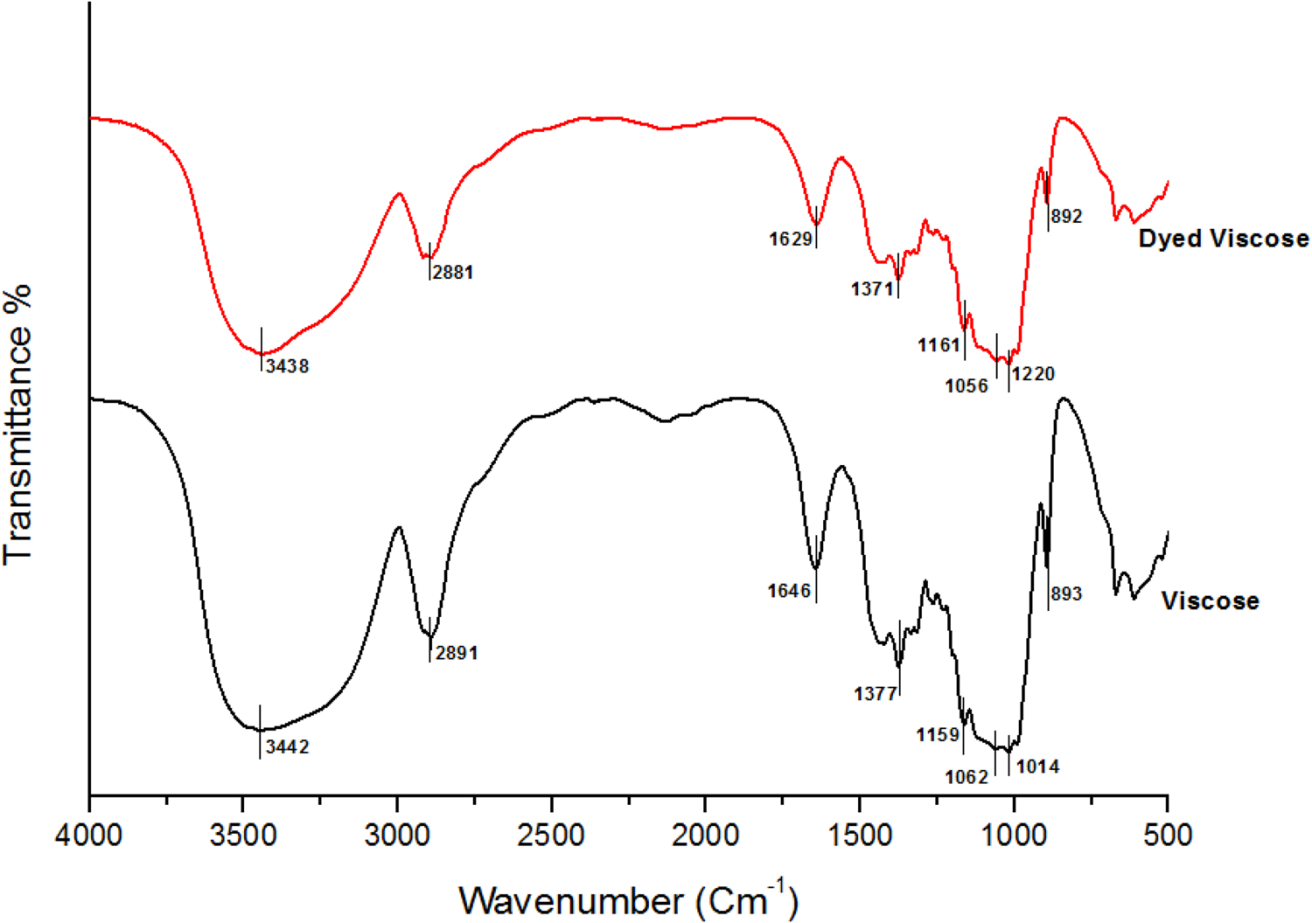
The FTIR spectra of undyed as well as dyed viscose fabrics.

The FTIR spectroscopy was adopted to attest the substantivity of viscose fabric to BTWE. The FTIR spectra of the undyed as well as dyed viscose fabrics were presented in Fig. 4. Being cellulosic in nature, the FT-IR spectrum of the untreated viscose sample has characteristic bands associated with the hydroxyl (O–H), the C–O, and the C–O–C bonds within the macromolecular structure of the fabric^77^. In Fig. 5, the broad band ranged between 3000 and 3600 cm⁻^1^ was attributed to the stretching vibration of hydrogen-bonded O–H groups. The band at 2891 cm⁻^1^ is due to C–H stretching and CH_2_ deformation vibrations. Additionally, bands associated with the C–O–C and C–O stretching vibrations appeared at 1159 and 1014 cm ^−1^, respectively. The OH stretching vibration of the absorbed water was observed as a strong band at 1646 cm^−1^. The sharp band with medium intensity at 1377 cm^−1^ belongs to the C–H bending vibration. A relatively strong band distinguishing the cellulosic substance for the C–O–C stretching of cellulose ring appeared at 893 cm^−1^ ^78,79^. The FTIR spectrum of the dyed viscose fabric exhibited similar pattern to that of the undyed sample with slight deviation in the wave numbers of the characteristics bands. It is clear from Fig. 8 that the intensity of the broad band around 3440 cm^−1^ decreased remarkably upon dyeing of viscose fabric with BTWE. This is an evidence of the formation of hydrogen bonds between hydroxyl groups in viscose fabric and the main colorants in the BTWE, which contain polar hydroxyl and carboxylic groups^80^.

**Fig. 9:**
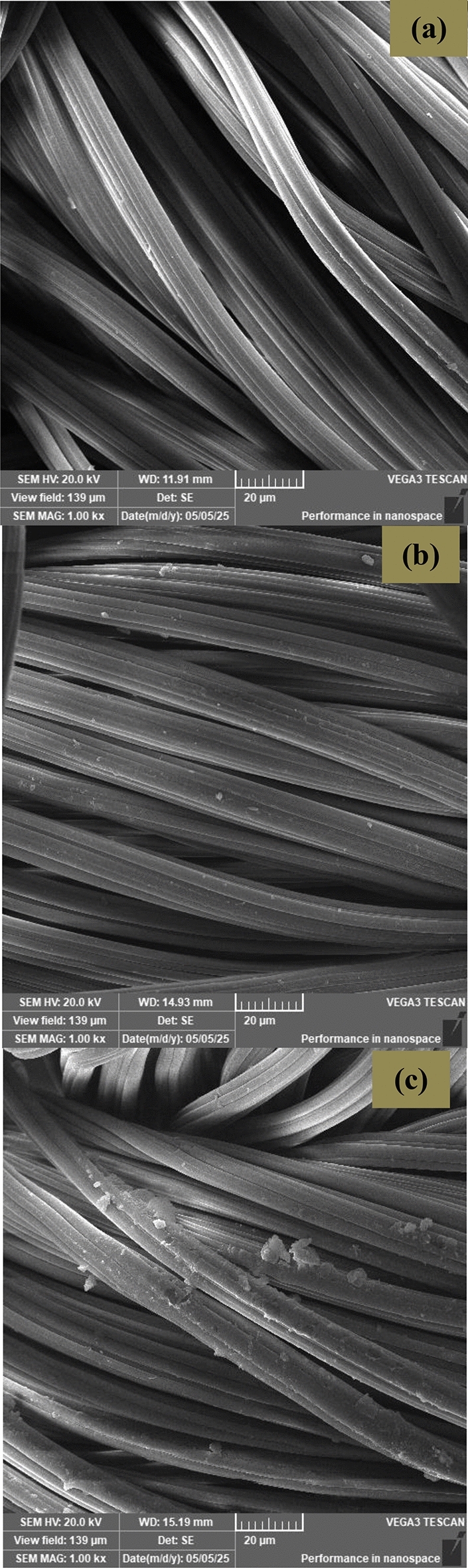
Morphological structure of viscose fabrics: (**a**) untreated, (**b**) dyed using BTWE, and (**c**) dyed using BTWE in the presence of ZnO NPs.

The surface morphology of the untreated viscose fabric as well as its corresponding dyed sample using BTWE in the presence and absence of ZnO NPs was investigated using a high- resolution scanning electron microscope (Fig. 9a–c). The scanning electron micrograph of the untreated fabric (Fig. 9a) parades the usual longitudinal fibril structure of viscose fibers with their smooth topography. Figure 9b elucidated that the fiber surface of the dyed sample looks similar to the untreated one, while the micrograph of viscose fabric dyed in the presence of ZnO NPs demonstrated coating of its surface with the metal oxide nanoparticles (Fig. 9c). No deteriorating action on the fibers’ morphology was detected in the dyed fabrics either in the presence or absence of ZnO NPs.

### Fabric properties

The effects of dyeing viscose fabric with BTWE in the presence and absence of ZnO NPs on its resistance to UV rays, tensile strength, and elongation at break were monitored, and the results were tabulated in Table 13. It is clear from this table that the undyed fabric has no resistance to the UV rays (UPF = 3). Dyeing of viscose fabric with BTWE increased its UPF to 10.0, which indicated that the dyed fabric is not UV-protective. This increase in the UPF of the dyed sample can be rationalized in terms of the imparted color to the dyed fabric, which enhanced its ability to absorb, rather than transmit; the UV rays^81^. Slight increase in the UPF of viscose fabric dyed with BTWE in the presence of ZnO NPs (UPF = 12.6). The limited improvement in the UPF of the ZnO NPs-treated fabrics may be due to ease of penetration of ZnO NPs into the fibers’ interiors, rather than accumulation on the fibers’ surfaces. Consequently, the surface of the dyed fabrics did not acquire adequate resistance to the UV rays. This hypothesis is supported by the earlier finding of Kathirvelu et al*.*^82^. This means that the dyed fabric cannot be used in the protection of human skin from the harmful effects of UV radiation, which limits its use in sunny weather for a long time^83^. Future work will be directed to enhance the UPF of the dyed fabric using the proper reagents, such as UV absorbers. The data in Table 13 also revealed that dyeing of viscose fabric with BTWE resulted in limited changes in its tensile strength and elongation at break, indicating that the dyed fabrics maintain adequate strength and flexibility.

**Table 13:** Fabric properties of viscose fabrics dyed with BTWE in the presence and absence of ZnO NPs.

Viscose fabric	UPF	Tensile strength (Kgf)	Elongation at break (%)
Undyed	3.0 ± 0.05	24.4 ± 0.04	32.0 ± 0.05
Dyed with BTWE	10.0 ± 0.06	23.7 ± 0.06	33.4 ± 0.06
Dyed in the presence of ZnO NPs	12.6 ± 0.08	23.5 ± 0.05	33.1 ± 0.05

Compared with other plant-derived colorants from hibiscus, red cabbage that have limited pH stability and poor light fastness, BTWE is relatively photo-stable by virtue of tannins which have distinguished oxidative stability^84^. Moreover, the combined coloration and functional properties of BTWE make it more appropriate for dyeing and finishing of textile substrates.

## Conclusion

Black tea waste extract (BTWE) demonstrated adequate substantivity towards viscose fabric at the proper dyeing conditions. The maximum dyeability was attained using 4% BTWE at 45 °C and pH 3 for 60 min. The relatively low dyeing temperature makes the proposed method a water- and energy-saving process for dyeing of viscose fabrics. The K/S of the dyed fabric was further increased upon dyeing in the presence of ZnO NPs. The dyed samples exhibited very good to excellent fastness properties against perspiration in acid medium, crocking, and light, reduced to good colorfastness against washing and perspiration in alkaline medium. Unlike the untreated viscose fabric, the antimicrobial activity of the dyed sample against Gram-positive, Gram-negative, and fungi was highly improved, up to growth reduction percentages of 91.67% in the case of *S. aureus*, 85.82% in the case of *C. albicans*, and 78.07% in the case of *E. coli*. Dyeing of viscose fabrics in the presence of ZnO NPs further enhanced the antimicrobial activity up to 98.88%. The synergism of the antimicrobial and antioxidant activities of the dyed viscose fabrics makes them appropriate for the production of protective clothing, such as medical textiles. This work proposed a method of proper utilization of plant waste material in a cheap sustainable eco-friendly method for dyeing and functional finishing of viscose fabrics without deterioration in the fibers’ strength. According to the findings, mixing ZnO nanoparticles with black tea waste extract offers a viable, environmentally friendly strategy for creating functional fabrics with health-protective characteristics, promoting the ideas of the circular economy and green chemistry in textile coloration. The durability of the dyed fabrics in terms of their microbial resistance is inadequate, suggesting their use in disposable medical textiles or in textile products that are not usually washed, such as tapestry. Our near future work will focus on scaling up the dyeing of viscose with BTWE, along with addressing the sustainability and cost-effectiveness of the entire process. An important challenge which should be addressed in future work is to use a dyeing bath of lower acidity for real-world implementation.

## Supplementary Information

Supplementary Information.

## Data Availability

The authors declared that the data supporting the findings of this study are available in the submitted manuscript.
