# Supplementary figures and images for: Synergetic effect of Camellia sinensis waste extract and zinc oxide nanoparticle for improving performance and appearance attributes of viscose fabrics
Source: Sci Rep. 2026 Mar 27;16:10917. doi: 10.1038/s41598-026-42384-4 (PMC13039887; doi:10.1038/s41598-026-42384-4)

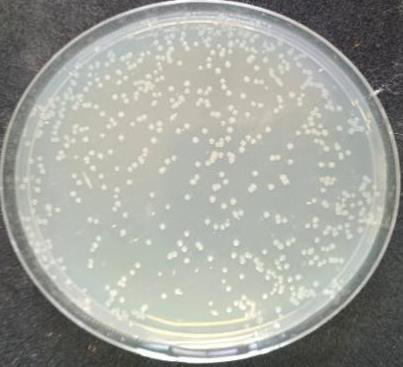

Supplement: Supplementary file 1 — Supplementary Information. [file 41598_2026_42384_MOESM1_ESM.zip › vertopal_806ea11db7cb4394a1d18243abfab664/media/image12.png]

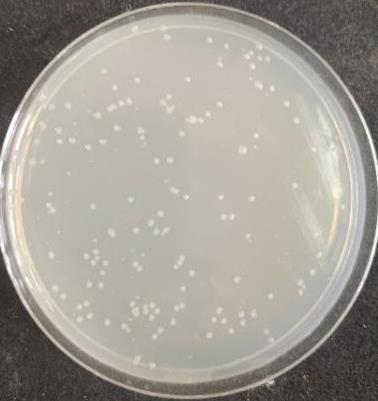

Supplement: Supplementary file 1 — Supplementary Information. [file 41598_2026_42384_MOESM1_ESM.zip › vertopal_806ea11db7cb4394a1d18243abfab664/media/image9.png]

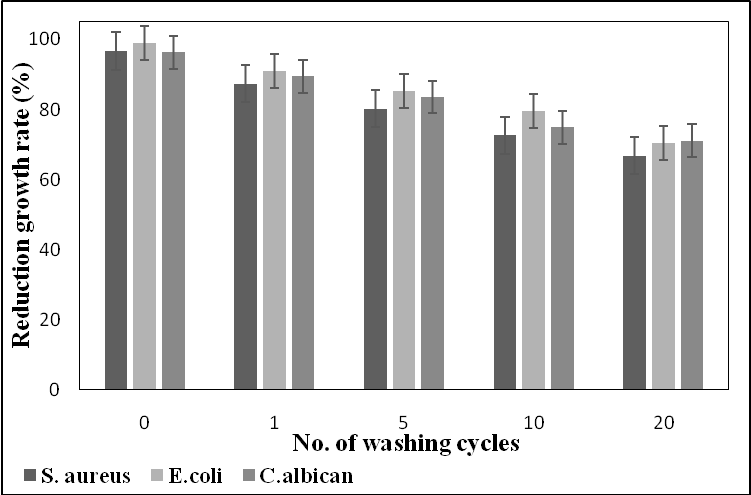

Supplement: Supplementary file 1 — Supplementary Information. [file 41598_2026_42384_MOESM1_ESM.zip › vertopal_806ea11db7cb4394a1d18243abfab664/media/image19.png]

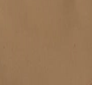

Supplement: Supplementary file 1 — Supplementary Information. [file 41598_2026_42384_MOESM1_ESM.zip › vertopal_806ea11db7cb4394a1d18243abfab664/media/image5.png]

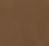

Supplement: Supplementary file 1 — Supplementary Information. [file 41598_2026_42384_MOESM1_ESM.zip › vertopal_806ea11db7cb4394a1d18243abfab664/media/image7.png]

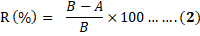

Supplement: Supplementary file 1 — Supplementary Information. [file 41598_2026_42384_MOESM1_ESM.zip › vertopal_806ea11db7cb4394a1d18243abfab664/media/image2.png]

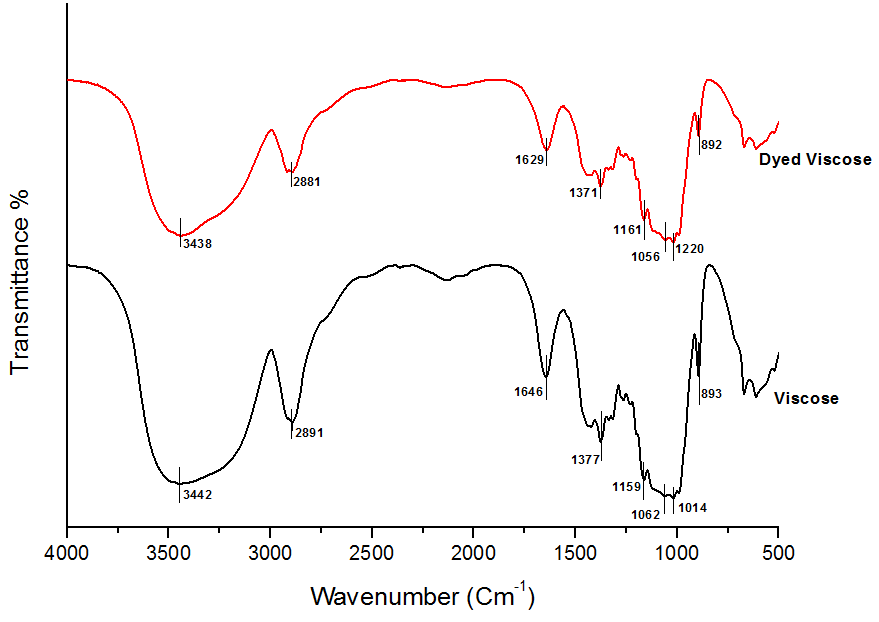

Supplement: Supplementary file 1 — Supplementary Information. [file 41598_2026_42384_MOESM1_ESM.zip › vertopal_806ea11db7cb4394a1d18243abfab664/media/image20.png]

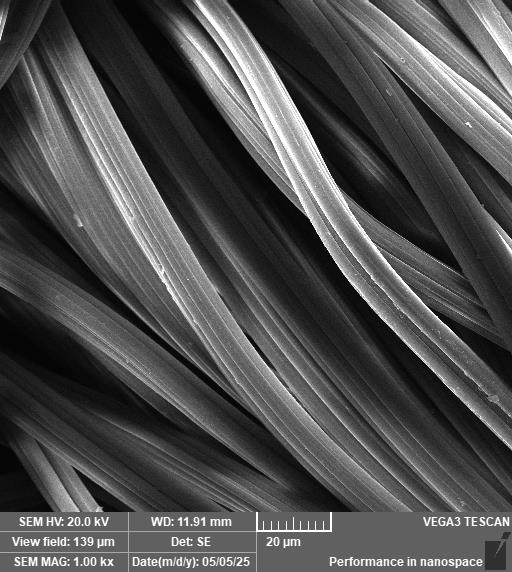

Supplement: Supplementary file 1 — Supplementary Information. [file 41598_2026_42384_MOESM1_ESM.zip › vertopal_806ea11db7cb4394a1d18243abfab664/media/image21.png]

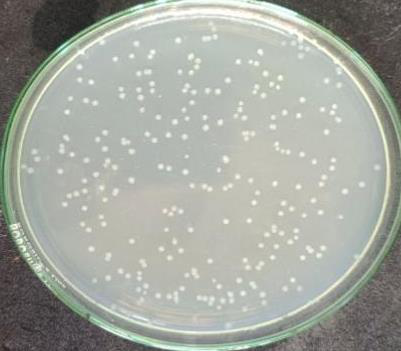

Supplement: Supplementary file 1 — Supplementary Information. [file 41598_2026_42384_MOESM1_ESM.zip › vertopal_806ea11db7cb4394a1d18243abfab664/media/image15.png]

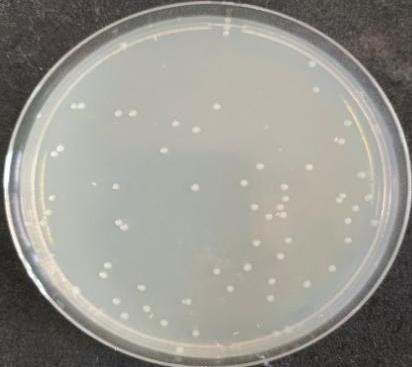

Supplement: Supplementary file 1 — Supplementary Information. [file 41598_2026_42384_MOESM1_ESM.zip › vertopal_806ea11db7cb4394a1d18243abfab664/media/image13.png]

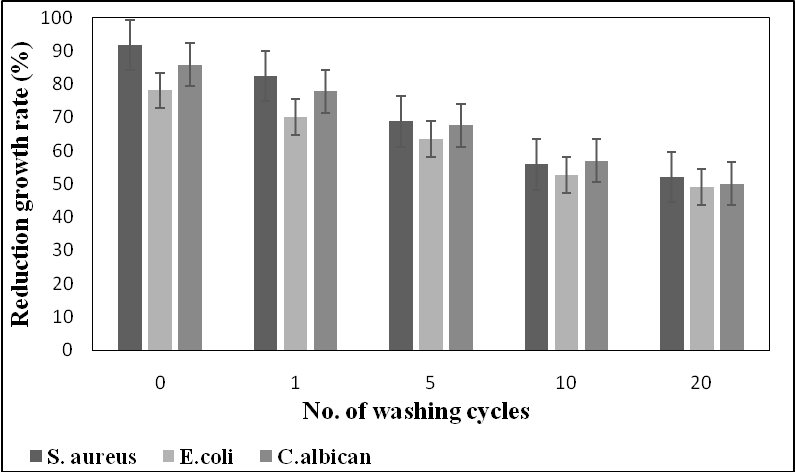

Supplement: Supplementary file 1 — Supplementary Information. [file 41598_2026_42384_MOESM1_ESM.zip › vertopal_806ea11db7cb4394a1d18243abfab664/media/image18.png]

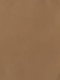

Supplement: Supplementary file 1 — Supplementary Information. [file 41598_2026_42384_MOESM1_ESM.zip › vertopal_806ea11db7cb4394a1d18243abfab664/media/image6.png]

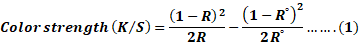

Supplement: Supplementary file 1 — Supplementary Information. [file 41598_2026_42384_MOESM1_ESM.zip › vertopal_806ea11db7cb4394a1d18243abfab664/media/image1.png]

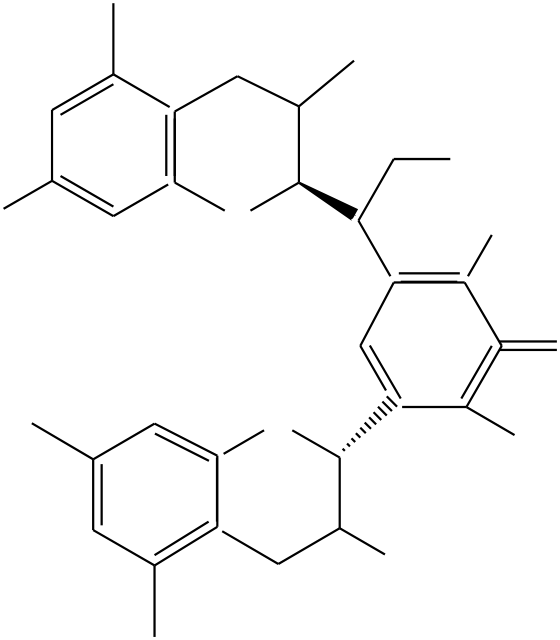

Supplement: Supplementary file 1 — Supplementary Information. [file 41598_2026_42384_MOESM1_ESM.zip › vertopal_806ea11db7cb4394a1d18243abfab664/media/image4.png]

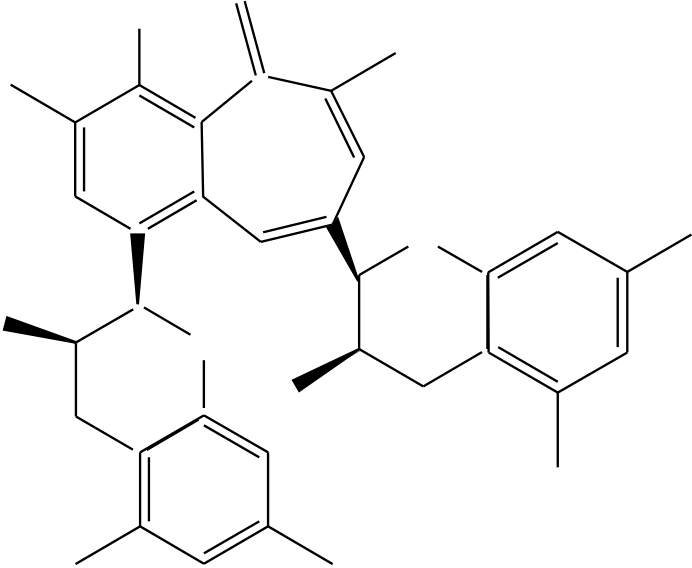

Supplement: Supplementary file 1 — Supplementary Information. [file 41598_2026_42384_MOESM1_ESM.zip › vertopal_806ea11db7cb4394a1d18243abfab664/media/image3.png]

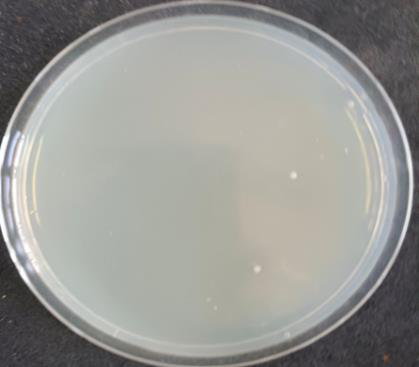

Supplement: Supplementary file 1 — Supplementary Information. [file 41598_2026_42384_MOESM1_ESM.zip › vertopal_806ea11db7cb4394a1d18243abfab664/media/image14.png]

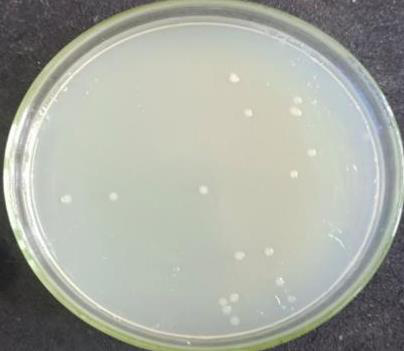

Supplement: Supplementary file 1 — Supplementary Information. [file 41598_2026_42384_MOESM1_ESM.zip › vertopal_806ea11db7cb4394a1d18243abfab664/media/image16.png]

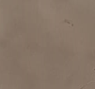

Supplement: Supplementary file 1 — Supplementary Information. [file 41598_2026_42384_MOESM1_ESM.zip › vertopal_806ea11db7cb4394a1d18243abfab664/media/image8.png]

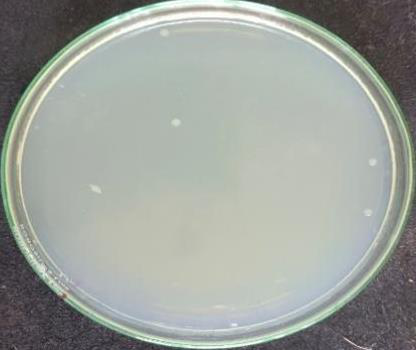

Supplement: Supplementary file 1 — Supplementary Information. [file 41598_2026_42384_MOESM1_ESM.zip › vertopal_806ea11db7cb4394a1d18243abfab664/media/image17.png]

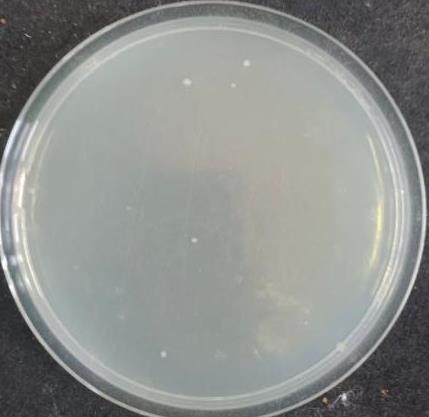

Supplement: Supplementary file 1 — Supplementary Information. [file 41598_2026_42384_MOESM1_ESM.zip › vertopal_806ea11db7cb4394a1d18243abfab664/media/image11.png]

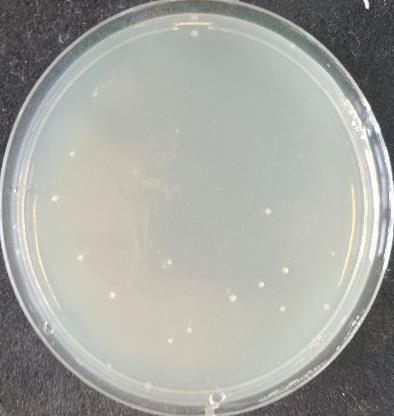

Supplement: Supplementary file 1 — Supplementary Information. [file 41598_2026_42384_MOESM1_ESM.zip › vertopal_806ea11db7cb4394a1d18243abfab664/media/image10.png]

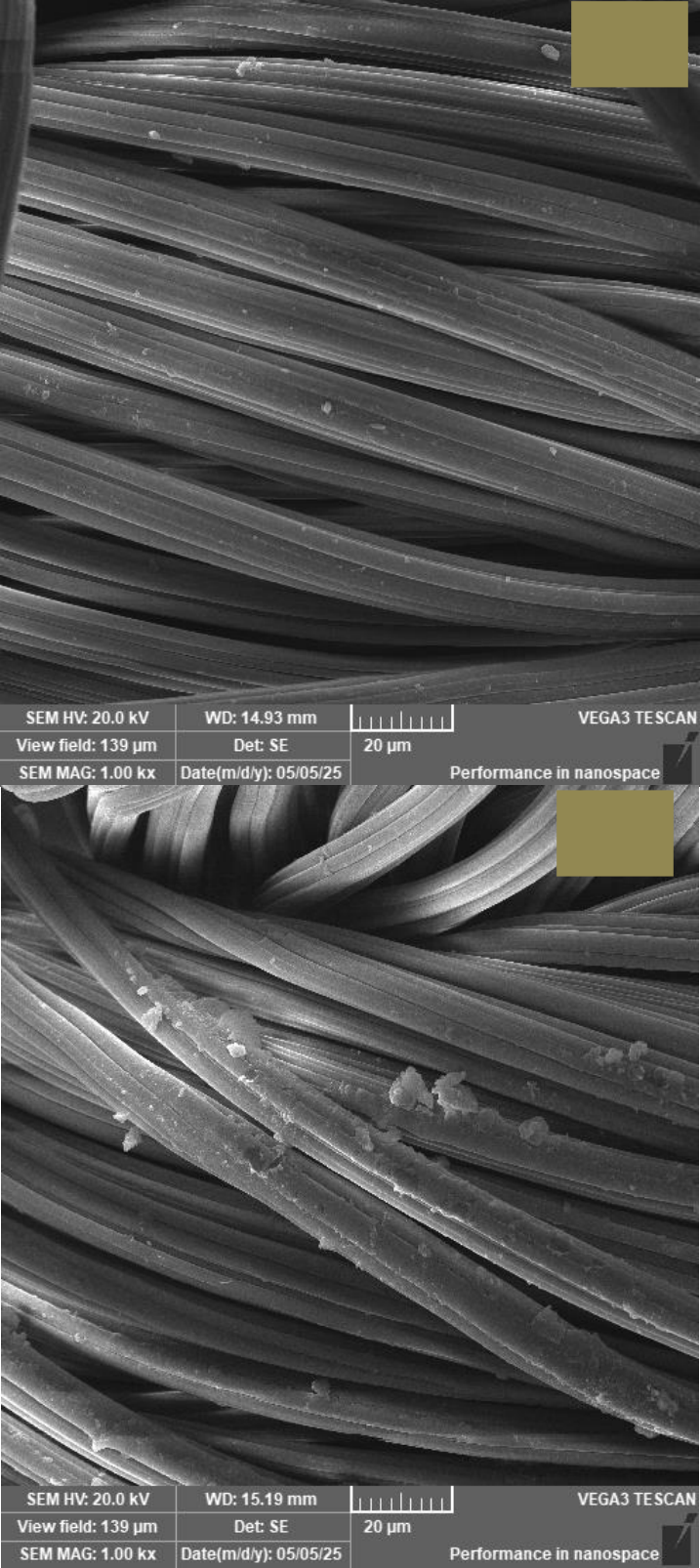

Supplement: Supplementary file 1 — Supplementary Information. [file 41598_2026_42384_MOESM1_ESM.zip › vertopal_806ea11db7cb4394a1d18243abfab664/media/image22.png]
